# The consensus sequence of *FAMLF* alternative splice variants is overexpressed in undifferentiated hematopoietic cells

**DOI:** 10.1590/1414-431X20154430

**Published:** 2015-06-12

**Authors:** W.L. Chen, D.F. Luo, C. Gao, Y. Ding, S.Y. Wang

**Affiliations:** 1 Union Clinical Medical College, Fujian Medical University, Fuzhou, China; 2 Department of Hematology, Fujian Institute of Hematology, Fujian Provincial Key Laboratory on Hematology, Fujian Medical University Union Hospital, Fuzhou, China

**Keywords:** *FAMLF*, Gene expression, Leukemia, Real-time polymerase chain reaction, Alternative splicing

## Abstract

The familial acute myeloid leukemia related factor gene (*FAMLF*) was previously identified from a familial AML subtractive cDNA library and shown to undergo alternative splicing. This study used real-time quantitative PCR to investigate the expression of the *FAMLF* alternative-splicing transcript consensus sequence (*FAMLF-CS*) in peripheral blood mononuclear cells (PBMCs) from 119 patients with *de novo* acute leukemia (AL) and 104 healthy controls, as well as in CD34^+^cells from 12 AL patients and 10 healthy donors. A 429-bp fragment from a novel splicing variant of *FAMLF* was obtained, and a 363-bp consensus sequence was targeted to quantify total *FAMLF* expression. Kruskal-Wallis, Nemenyi, Spearman's correlation, and Mann-Whitney U-tests were used to analyze the data. *FAMLF-CS* expression in PBMCs from AL patients and CD34^+^ cells from AL patients and controls was significantly higher than in control PBMCs (P<0.0001). Moreover,*FAMLF-CS* expression in PBMCs from the AML group was positively correlated with red blood cell count (r_s_ *=*0.317, P*=*0.006), hemoglobin levels (r_s_ *=*0.210, P=0.049), and percentage of peripheral blood blasts (r_s_ *=*0.256, P=0.027), but inversely correlated with hemoglobin levels in the control group (r_s_ *=*–0.391, P<0.0001). AML patients with high CD34^+^ expression showed significantly higher*FAMLF-CS* expression than those with low CD34^+^ expression (P=0.041). Our results showed that*FAMLF* is highly expressed in both normal and malignant immature hematopoietic cells, but that expression is lower in normal mature PBMCs.

## Introduction

Familial acute leukemia is an inherited malignancy whose genetic pool offers an effective research avenue to elucidate disease pathogenesis through the investigation of acute leukemia (AL) pedigrees with high incidence (1–5). We previously identified an acute myeloid leukemia (AML) pedigree in a village in Fujian Province, China (6,7), from which we constructed a subtracted cDNA library using super-switching mechanism at RNA termini polymerase chain reaction (SMART-PCR) and suppression subtractive hybridization (8). Subsequent sequence analysis revealed that 11 expressed sequence tags (ESTs) did not match any known sequences in the GenBank/EMBL database. Using SMART rapid amplification of cDNA ends, we obtained the complete cDNA sequence for one selected EST (GenBank accession no. CV973101.1) (9), and derived the full nucleotide sequence of a novel gene transcript (GenBank accession no. EF413001.1) consisting of two exons. This gene, named *Homo sapiens* familial acute myeloid leukemia related factor (*FAMLF*), is localized to chromosome 1q32.1. Bioinformatics analysis predicted that *FAMLF* may participate in the transduction of cellular messages and be associated with cell proliferation or apoptosis (9).

Recent data from genome-wide studies have suggested that more than 90% of human genes undergo alternative splicing (10). Indeed, evidence has been found for the existence of alternative *FAMLF*splicing variants, with the retrieval of a *FAMLF* transcript from the NCBI database (GenBank accession no. NR_040073.1) containing the identical nucleotide sequence to EF413001.1 at position 1–365 bp. This transcript has three exons and was defined as a long non-coding RNA. Based on the position of the second exon of NR_040073.1, we speculated that 1–363 bp may be the consensus sequence of *FAMLF* RNAs. We confirmed this by reverse transcription (RT)-PCR. We have also shown that CV973101.1 was highly expressed in the affected family member of the familial AML pedigree, but remained at a low level in the unaffected relative (8). The BLAST (Basic Local Alignment Search Tool) search identified that the CV973101.1 sequence is located at 25–279 bp of the alternative *FAMLF* splicing transcript (EF413001.1 or NR_040073.1). We propose that it is of interest to explore the 1–363 bp sequence, and to verify the expression of*FAMLF* splicing variants consensus sequence in AL.

Previously, we examined the expression of EF413001.1 in peripheral blood mononuclear cells (PBMCs) from 23 patients with *de novo* AML, 23 controls, and another nine healthy individuals from the familial AML pedigree using semi-quantitative PCR. EF413001.1 was shown to be overexpressed in patient PBMCs compared with those from controls (9). However, EF413001.1 expression does not represent the complete*FAMLF* expression, because research has shown that many alternative splice variants from the same gene have different expression patterns (11,12). Hence, the total *FAMLF* expression in AL patients and controls remains to be determined. Moreover, it will be worthwhile to detect the expression of the *FAMLF* alternative-splicing transcript consensus sequence (*FAMLF-CS*; 1–363 bp) to evaluate the possible functional involvement of the gene in AL. Thus, the present study used real-time quantitative (RQ)-PCR to examine the total *FAMLF* RNA expression using primers targeting the consensus sequence in PBMCs and CD34^+^ cells from AL patients and healthy controls. High expression of *FAMLF* was detected in both normal and malignant naive hematopoietic cells.

## Material and Methods

### Sample collection

A total of 233 subjects were enrolled in the present study, including 119 patients with *de novo* AL, 104 healthy individuals, and 10 healthy pregnant women. The patients were selected from the Department of Hematology, Fujian Medical University Union Hospital, Fuzhou, China, from September 2010 to April 2012. AL diagnosis and classification were based on morphologic, cytochemical, and immunophenotypic criteria proposed by the French-American-British Committee (13,14). Samples collected from AL patients were analyzed at diagnosis. The healthy individuals in this study were from the Medical Center of Fujian Medical University Union Hospital. The pregnant women were from the Obstetrical Department, Fujian Provincial Maternal and Child Health Care Hospital. Subjects provided their written informed consent for the use of blood samples and access to clinical information. The procedures for our study were conducted in accordance with the guidelines of the Medical Ethics Committees of the Health Bureau of Fujian Province, China.

Peripheral blood (10 mL) from AL patients (n=119) or healthy individuals (n=104), and bone marrow (10 mL) aspirated from the posterior iliac crest of AL patients (n=12) were collected in sterile EDTA tubes. Umbilical cord blood (UCB; n=10) was obtained after full-term normal vaginal deliveries from the healthy pregnant women and placed in a 50-mL sterile centrifuge tube containing 400 U of preservative-free heparin. PBMCs, bone marrow mononuclear cells (BMMCs), and umbilical cord blood mononuclear cells (UCBMCs) were isolated by Ficoll-Hypaque density gradient centrifugation (400*g*, 20 min), and washed twice with phosphate-buffered saline.

CD34^+^ cells from healthy donors were separated from UCBMCs, and those of AL patients separated from BMMCs using fluorescence-activated cell sorting. UCBMCs and BMMCs were labeled with phycoerythrin (PE)-conjugated mouse anti-human CD34^+^ monoclonal antibody (eBioscience, USA) according to the manufacturer's recommendations. Cell sorting was performed using a FACStar^PLUS^ cell sorter (Becton Dickinson, USA) and the threshold for selection of CD34^+^ cells was based on the comparison with a PE-conjugated isotype control antibody (IgG_1_, eBioscience). Purity of the separated CD34^+^ cells was assessed by analysis of an aliquot of sorted cells and was routinely greater than 99%. PBMC and CD34^+^ cell samples were stored at –70°C and used for RNA extraction.

### RNA extraction and reverse transcription

Total RNA was extracted from PBMCs and CD34^+^ cells using Trizol reagent (Invitrogen, USA) according to the manufacturer's instructions. The integrity of the RNA samples was determined by electrophoresis through a denaturing agarose gel and staining with ethidium bromide. The 18S and 28S RNA bands were visualized in ultraviolet light. Spectrophotometric readings at wavelengths of 260 and 280 nm were obtained to evaluate the quantity and purity of the isolated RNA.

The RNA template was then prepared and transcribed into first-strand cDNA using a RevertAid™ First Strand cDNA Synthesis Kit (Fermentas, Canada). Briefly, 2 μg of total RNA was incubated at 25°C for 5 min, at 42°C for 60 min, then at 70°C for 5 min with 0.2 μg random hexamer primer, 200 U RevertAid™ M-MuLV reverse transcriptase, 20 U RiboLock™ RNase inhibitor, 2 μL 10 mM dNTPs, and 4 μL 5× reaction buffer (250 mM Tris-HCl, 250 mM KCl, 20 mM MgCl_2_, and 50 mM DTT, pH 8.3) in a total volume of 20 μL. The cDNA was stored at –20°C.

### PCR

To detect alternative *FAMLF* splicing transcripts, PCR was conducted in a 25 μL solution containing 2 μL cDNA template, 12.5 μL 2× DreamTaq™ Green PCR Master Mix (Fermentas), 1 μL 10 μmol/L *FAMLF*primer pairs (forward primer: 5'-CAGGAGCAAGGGATGTCTG-3', reverse primer: 5'-CCACCAAAACTGATGAAATAGC-3'), and 9.5 μL H_2_O. PCR reactions were carried out at 94°C for 5 min, then 30 cycles of 94°C for 30 s, 57°C for 30 s, and 72°C for 2 min, followed by a final extension at 72°C for 10 min. PCR products were analyzed by electrophoresis on a 1% agarose gel containing ethidium bromide and sent to Shanghai Invitrogen Biotechnology Co., Ltd. (China) for DNA sequence analysis.

### Real-time quantitative PCR (RQ-PCR)

The following *FAMLF-CS*-specific primers were used to amplify a 147-bp product by RQ-PCR: forward primer: 5′-ACCGTTTTGAAATTAGATCC-3′ (exons 1/2, nt position: 191–210, GenBank accession no. EF413001.1/ NR_040073.1), reverse primer: 5′-CCACCAACCAAGCTACTCAC-3′ (exon 2, nt position: 337–318, GenBank accession no. EF413001.1/NR_040073.1).

The endogenous control gene *β-actin* was used to check the quality of the RNA samples and to normalize the variations from different samples. *β-actin*-specific primers for the amplification of a 220-bp product were as follows: forward primer: 5′-AGTGTGACGTGGACATCCGCAAAG-3′ (exon 5, nt position: 935–958, GenBank accession no. NM_001101.3), and reverse primer: 5′-ATCCACATCTGCTGGAAGGTGGAC-3′ (exon 6, nt position: 1154–1131, GenBank accession no. NM_001101.3). Shanghai Invitrogen Biotechnology Co., Ltd. synthesized all primers.

Expression levels of the target (*FAMLF-CS*) and reference genes (*β-actin*) were determined by SYBR Green I RQ-PCR assays of 96-well plates. All samples were run in triplicate on an ABI 7500 Real-Time PCR System (Applied Biosystems, USA). PCR was performed in a total volume of 25 μL containing 1 μL cDNA template, 12.5 μL 2× FastStart Universal SYBR Green Master Mix (Roche, USA), 0.6 μL 10 μmol/L *FAMLF-CS* or*β-actin* primer pairs, and 10.9 μL H_2_O. *FAMLF-CS* and *β-actin*were amplified in separate wells. The reaction protocol involved initial heating at 50°C for 2 min and 95°C for 10 min, followed by 40 cycles of 95°C for 15 s and 55°C for 1 min. In each RQ-PCR set-up, two control samples were used to monitor the variation between plates, which was controlled to be less than 5% in this study. Three template-free samples were included as negative controls. All replicates within 0.5 C_T_ of each other were acceptable. Additionally, PCR product specificity was confirmed by melting curve analysis and agarose electrophoresis. PCR products were sent to Shanghai Invitrogen Biotechnology Co., Ltd. for DNA sequence analysis.

Data were analyzed using the comparative C_T_ method using the 7500 Fast System SDS software (Applied Biosystems) (15). The relative quantitation of *FAMLF-CS*expression was calculated as 2^-δCt^, where δC_T=_(C_T *FAMLF-CS*_– C_T *β-actin*_). Before the δC_T_ calculation was performed,*FAMLF-CS* and *β-actin* values were averaged separately. Medians and interquartile ranges were calculated for each group as individual data points using 2^-δC^.

### Statistical analyses

Data analysis was performed using SPSS version 15 (SPSS Inc., USA). Quantitative variables are reported as medians and (interquartile) ranges, and qualitative variables are reported as numbers and percentages. The Kruskal-Wallis test was used to determine if the difference in *FAMLF-CS* expression among the four groups (PBMCs from AL patients, PBMCs from healthy individuals, CD34^+^ cells from AL patients, and CD34^+^cells from healthy donors) was statistically significant. If so, the Nemenyi test was used to analyze the difference between the two groups. These statistical analyses were also used to analyze differences in*FAMLF-CS* expression among the AML, acute lymphocytic leukemia (ALL), and control groups. Spearman's correlation test analyzed the correlation between *FAMLF-CS* expression and hematological parameters. Finally, the Mann-Whitney U-test was used to analyze the differential expression of *FAMLF-CS* in PBMCs from AL patients with high and low CD34^+^ expression. A P value <0.05 was considered to be statistically significant.

## Results

### *FAMLF* has a novel splicing variant and 1–363 bp is the consensus sequence of *FAMLF* RNAs

**Figure 1:**
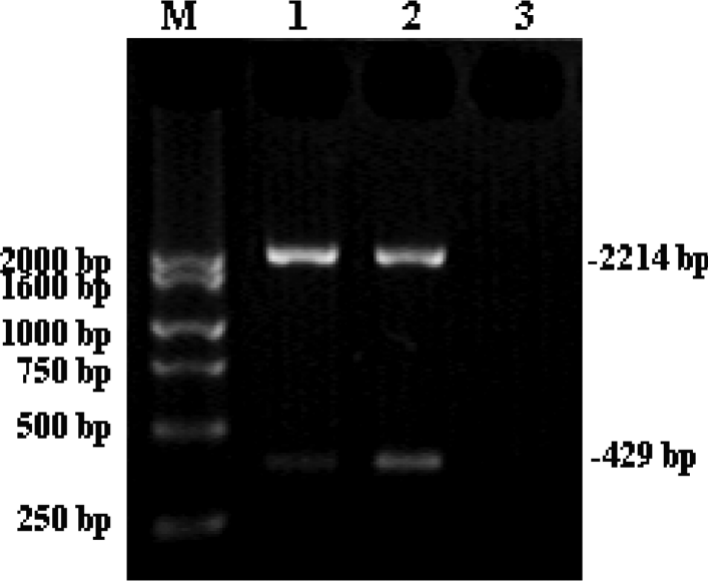
RT-PCR amplification using cDNA templates from two acute leukemia (AL) patients revealed an expected 2214-bp band and an unexpected 429-bp band representing differential splicing. Lanes: M: molecular size markers; 1 and 2: the two AL patients; 3: negative control.

**Figure 2:**
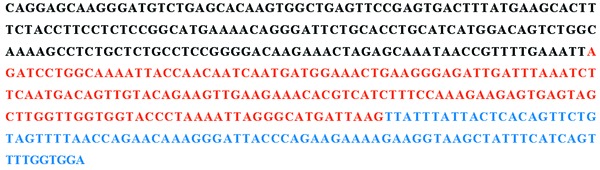
Sequence acquisitions of 429-bp fragments showing inclusion of the consensus sequence. Black, red, and blue type indicate individual exons. Black and red represent part of the consensus sequence (338 bp).

RT-PCR was performed to confirm our speculation about the *FAMLF*consensus sequence. Two peripheral blood samples from AL patients were amplified by RT-PCR and a specific band of expected size (2214 bp) was obtained, as well as an unexpected 429-bp band (Figure 1). Sequencing revealed that the unexpected fragment shared 338-bp of its sequence with that of the two known *FAMLF* splicing variants (nt position: 26–363 bp) (Figure 2). This represented a partial sequence of a novel*FAMLF* alternative splicing transcript, and confirmed the 1–363 bp sequence as the consensus sequence of*FAMLF* alternative splicing variants. We therefore designed primers to target the consensus sequence for quantitative *FAMLF*total RNA expression.

### *FAMLF-CS* is overexpressed in the PBMCs of AL patients and CD34^+^ cells of AL patients and healthy donors

**Figure 3:**
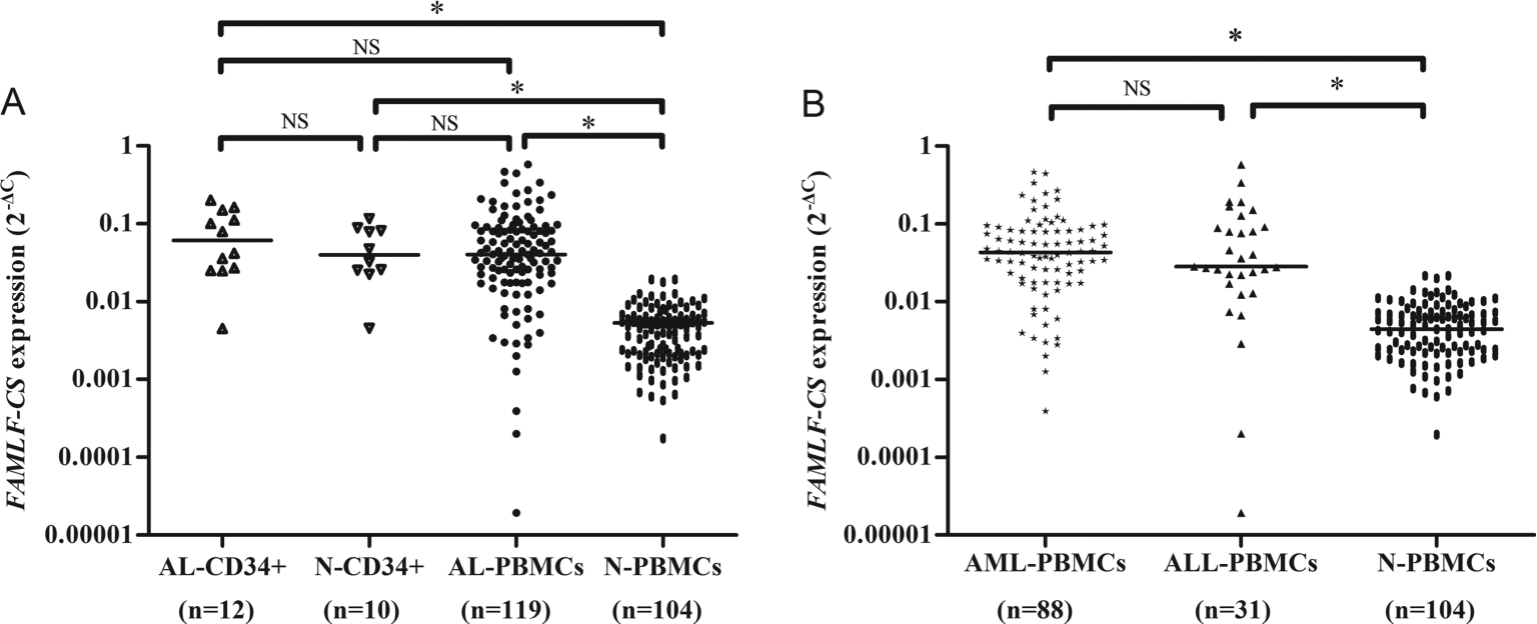
RQ-PCR analysis of *FAMLF-CS* expression in CD34^+^ cells and peripheral blood mononuclear cells (PBMCs) from acute leukemia (AL) patients and normal healthy controls (N). *A*, Expression of *FAMLF-CS* in CD34^+^ cells and PBMCs from AL patients and normal healthy controls. *B*, Expression of*FAMLF*-*CS* in PBMCs from patients with acute myeloid leukemia (AML), acute lymphocytic leukemia (ALL), and normal healthy controls (N). Data of the relative quantitation of*FAMLF-CS* expression are reported as 2^-δC^. Horizontal bars represent the median*FAMLF-CS* expression for each group. *P<0.05 between groups (Nemenyi test). AL-CD34^+^: CD34^+^ cells from AL patients; N-CD34^+^: CD34^+^ cells from normal healthy controls; AL-PBMCs: PBMCs from AL patients; N-PBMCs: PBMCs from normal healthy controls; AML-PBMCs: PBMCs from AML patients; ALL-PBMCs: PBMCs from ALL patients; NS: not significant.

*FAMLF-CS* expression was investigated by RQ-PCR in PBMCs from 119 newly diagnosed patients with AL and 104 healthy donors, as well as in CD34^+^ cells from 12 AL patient bone marrow samples and 10 UCB samples from healthy donors. The general characteristics of AL patients and normal individuals are shown in Table 1.*FAMLF-CS* expression was observed in the PBMCs of all normal individuals, with a median relative value of 0.0044 (interquartile range, 0.0022–0.0070; Figure 3).

**Table:**
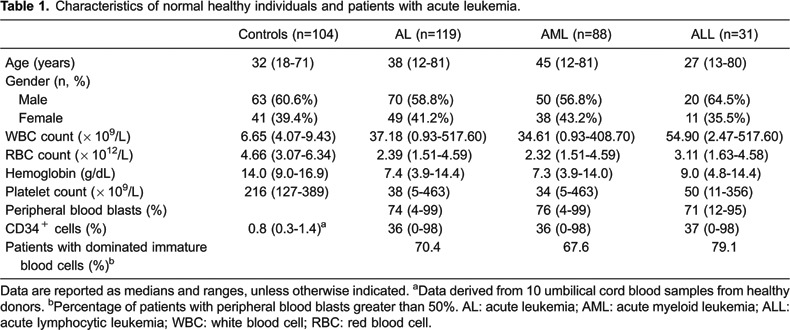

Of the 119 patients tested, 108 (90.8%) demonstrated PBMC*FAMLF-CS* expression levels that exceeded the median of normal individuals. The median *FAMLF-CS* expression in AL patient PBMCs was 0.0403 (interquartile range, 0.0177–0.0845; Figure 3A), representing an increase in expression of more than 9-fold compared with controls (P<0.0001; Figure 3A). No significant difference was observed between the AML group (n=88) and ALL group (n=31) (P*=*0.694; Figure 3B).

High expression of *FAMLF-CS* was also found in CD34^+^ cells from healthy donors, with a median level of 0.0400 (interquartile range, 0.0245–0.0834), and CD34^+^cells from AL patients, with a median level of 0.0608 (interquartile range: 0.0255–0.1410). A significant difference was detected in*FAMLF-CS* expression between PBMCs from normal individuals and CD34^+^ cells from healthy donors or AL patients (P<0.0001; Figure 3A). CD34^+^ cells from AL patients showed the highest median*FAMLF-CS* expression level, followed by PBMCs from AL patients, then CD34^+^ cells from healthy donors. However, no significant difference was observed between any two of these three groups (P>0.05, Figure 3A).

### *FAMLF-CS* expression is correlated with hematological parameters of AL patients and healthy controls

No correlation was observed between *FAMLF-CS* expression and white blood cell (WBC) count, red blood cell (RBC) count, or platelet count in the PBMCs from the healthy control group. However, an inverse correlation was observed between *FAMLF-CS* expression and hemoglobin levels (r_s_ *=*–0.391, P<0.0001; Table 2) in this group. By contrast, significant and positive correlations were observed between *FAMLF-CS* expression and RBC count (r_s_=0.225, P=0.025), and the percentage of peripheral blood blasts (r_s_=0.235, P=0.020) in AL PBMCs. However,*FAMLF-CS* expression did not significantly correlate with WBC count, hemoglobin levels, platelet count, or percentage of bone marrow-derived CD34^+^ cells (P>0.05; Table 2). Similarly, *FAMLF-CS* expression levels in the AML group were significantly correlated with RBC count (r_s_=0.317, P=0.006), hemoglobin levels (r_s_=0.210, P=0.049), and percentage of peripheral blood blasts (r_s_=0.256, P=0.027), but not with WBC count, platelet count, or percentage of CD34^+^ cells (P*>*0.05; Table 2). Additionally, no significant relationships were observed in the ALL group between *FAMLF-CS*expression and WBC count, RBC count, hemoglobin levels, platelet count, percentage of peripheral blood blasts, or percentage of CD34^+^cells (P*>*0.05; Table 2).

**Table:**
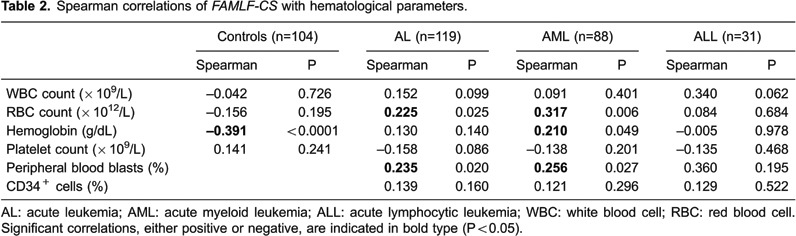

To further analyze the correlation between *FAMLF-CS* expression and the percentage of CD34^+^ cells, patients were divided into low and high expression groups using the median levels of percentage of CD34^+^ cells as cut-off points. AL patients with a high expression of CD34^+^ cells showed a trend toward a higher*FAMLF-CS* expression than those in the low expression group, although this difference was not significant (P=0.078, Table 3). AML patients with a higher percentage of CD34^+^ cells had a similarly, and significantly, higher*FAMLF-CS* expression compared with those patients with lower CD34^+^ expression (P=0.041, Table 3), but this significant difference was not found in the ALL group (P=0.905, Table 3).

**Table:**
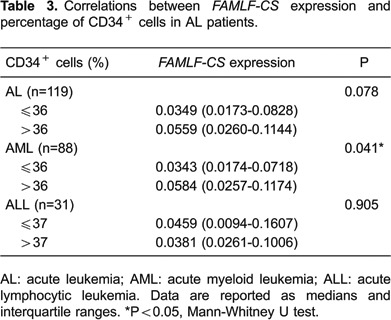

## Discussion

In this study, we obtained a 429-bp fragment from a novel splicing variant of*FAMLF*, and confirmed that the 1–363 bp sequence was the consensus sequence of known *FAMLF* splicing variants by comparing it with other *FAMLF* transcripts (EF413001.1 and NR_040073.1). To verify previous results, measure the total expression of different *FAMLF* splicing variants, and gain insight into*FAMLF* potential function in AL, we first examined*FAMLF-CS* expression in the PBMCs and CD34^+^cells from AL patients and healthy controls by RQ-PCR using primers targeting the consensus sequence.

Our findings demonstrated that *FAMLF-CS* expression was significantly higher in CD34^+^ cells from AL patients and healthy donors as well as in the PBMCs from AL patients compared with PBMCs from healthy individuals. CD34^+^ cells, including hematopoietic stem cells and early committed progenitors (16–18), were normal or malignant immature hematopoietic cells in this study. This compares with PBMCs from normal individuals, which are mature cells, while immature blood cells dominated the peripheral blood cells from 70.4% of AL patients. Therefore, our results indicated that*FAMLF-CS* is highly expressed in both normal and malignant immature hematopoietic cells, but is expressed at a low level in normal mature PBMCs.

We also found that AML patients with a higher expression of CD34^+^cells demonstrated higher *FAMLF-CS* expression than those with lower CD34^+^ expression, while *FAMLF-CS* was positively correlated with the percentage of peripheral blood blasts in AL patient PBMCs. Hence, the expression of *FAMLF-CS* appears to be associated with the differentiation status of hematopoietic cells involved in the study. Previous research revealed a substantial connection between hematopoietic cell differentiation and leukemogenesis or AL development (19–21), indicating that *FAMLF-CS* may participate in the development of leukemia through its role in hematopoietic cell differentiation.

Our study also revealed that the observed higher median expression level of*FAMLF-CS* in CD34^+^ cells from AL patients compared with those from healthy donors was not significantly greater, implying that*FAMLF* may not be defective in hematopoietic stem or progenitor cells. Thus, it is more likely that *FAMLF-CS* is associated with hematopoietic cell differentiation rather than leukemogenesis.

Our findings therefore provided evidence for an association between*FAMLF-CS* expression and hematopoietic cell differentiation, suggesting that the consensus sequence (1–363 bp) located at the 5' untranslated region (UTR) of EF413001.1 plays a pivotal role in the regulation of hematopoietic cell differentiation. Bernstein et al. (22) reported that the 5'-UTR of the platelet-derived growth factor B gene (PDGF2/c-*sis*) shows translational modulating activity during the megakaryocytic differentiation of K562 cells. Likewise, Fiaschi et al. (23) demonstrated that a relief of the inhibitory role of the 5'-UTR in the differentiated lineage process is responsible for the observed increase in acylphosphatase levels. Moreover, the 5'-UTRs of some genes are required for angiogenesis, hematopoiesis, and leukemogenesis (24,25). Because the 1–363 bp consensus sequence apparently plays a critical role in*FAMLF* control, it may be possible for future studies to use small interfering RNA to target this region with the aim of studying the function of*FAMLF*.

We additionally observed a positive correlation between *FAMLF-CS*expression and RBC count in the PBMCs from AML patients, but not in those from normal individuals. Moreover, *FAMLF-CS* expression was positively correlated with hemoglobin levels in the PBMCs from AML patients, but inversely correlated in the PBMCs from normal individuals. These results indicate that*FAMLF* may have various functions in leukemia and normal cells.

In summary, our study showed that *FAMLF-CS* was highly expressed in immature hematopoietic cells, and that the higher expression of*FAMLF-CS* in the PBMCs from AML patients was significantly associated with higher RBC count, hemoglobin levels, and percentage of peripheral blood blasts. AML patients with higher CD34^+^ cell expression also showed higher *FAMLF-CS* expression. Our results therefore provide evidence for an association between *FAMLF-CS* expression and hematopoietic cell differentiation, suggesting that *FAMLF-CS* may be involved in the development of leukemia. However, further investigations are necessary to corroborate this relationship and to clarify the underlying mechanisms responsible for the role of *FAMLF-CS* in hematopoietic cell differentiation.
